# Placental TLR recognition of salivary and subgingival microbiota is associated with pregnancy complications

**DOI:** 10.1186/s40168-024-01761-9

**Published:** 2024-03-26

**Authors:** Kazune Pax, Nurcan Buduneli, Murat Alan, Pinar Meric, Onder Gurlek, Shareef M. Dabdoub, Purnima S. Kumar

**Affiliations:** 1 Division of Oral Biosciences, College of Dentistry, The Ohio State University, Columbus, OH 43210 USA; 2 Faculty of Clinical Sciences, Department of Periodontology, Ege University, İzmir, Turkey; 3 Department of Obstetrics and Gynecology, Izmir Tepecik Training and Research Hospital, Tepecik, 35120 Izmir, Türkiye; 4 Department of Periodontics, Division of Biostatistics and Computational Biology, The University of Iowa School of Dentistry, Iowa City, IA 52242-1010 USA; 5 Department of Periodontics and Oral Medicine, School of Dentistry, University of Michigan, Ann Arbor, MI 48109 USA

**Keywords:** Pregnancy outcomes, Pre-term birth, Pre-eclampsia, Salivary bacteria, Placenta, Serum, Subgingival, DNA sequence analysis, Oral microbiome, Metagenomics

## Abstract

**Background:**

Pre-term birth, the leading cause of neonatal mortality, has been associated with maternal periodontal disease and the presence of oral pathogens in the placenta. However, the mechanisms that underpin this link are not known. This investigation aimed to identify the origins of placental microbiota and to interrogate the association between parturition complications and immune recognition of placental microbial motifs.

Video Abstract

**Methods:**

Saliva, plaque, serum, and placenta were collected during 130 full-term (FT), pre-term (PT), or pre-term complicated by pre-eclampsia (PTPE) deliveries and subjected to whole-genome shotgun sequencing. Real-time quantitative PCR was used to measure toll-like receptors (TLR) 1–10 expression in placental samples. Source tracking was employed to trace the origins of the placental microbiota.

**Results:**

We discovered 10,007 functionally annotated genes representing 420 taxa in the placenta that could not be attributed to contamination. Placental microbial composition was the biggest discriminator of pregnancy complications, outweighing hypertension, BMI, smoking, and maternal age. A machine-learning algorithm trained on this microbial dataset predicted PTPE and PT with error rates of 4.05% and 8.6% (taxonomy) and 6.21% and 7.38% (function). Logistic regression revealed 32% higher odds of parturition complication (95% CI 2.8%, 81%) for every IQR increase in the Shannon diversity index after adjusting for maternal smoking status, maternal age, and gravida. We also discovered distinct expression patterns of TLRs that detect RNA- and DNA-containing antigens in the three groups, with significant upregulation of TLR9, and concomitant downregulation of TLR7 in PTPE and PT groups, and dense correlation networks between microbial genes and these TLRs. 70–82% of placental microbiota were traced to serum and thence to the salivary and subgingival microbiomes. The oral and serum microbiomes of PTPE and PT groups displayed significant enrichment of genes encoding iron transport, exosome, adhesion, quorum sensing, lipopolysaccharide, biofilm, and steroid degradation.

**Conclusions:**

Within the limits of cross-sectional analysis, we find evidence to suggest that oral bacteria might translocate to the placenta via serum and trigger immune signaling pathways capable of inducing placental vascular pathology. This might explain, in part, the higher incidence of obstetric syndromes in women with periodontal disease.

**Supplementary Information:**

The online version contains supplementary material available at 10.1186/s40168-024-01761-9.

## Introduction

Pre-term birth, defined as any birth that occurs before 37 weeks of gestation, is the leading cause of neonatal mortality [1]. Premature babies often have more health problems than those born at full-term; including breathing issues, necrotizing enterocolitis, and intellectual and developmental disabilities [2]. Pre-term birth can be either spontaneous (unplanned) or medically indicated/induced (planned). While the exact cause of spontaneous preterm parturition is unknown, it is believed to be multifactorial, with infection and infection-driven inflammation as the leading factors [3]. 15% of pre-term births are also attributed to pre-eclampsia, a potentially life-threatening condition that is characterized by the onset of systemic hypertension and proteinuria during gestation. It has been postulated that the etiology of pre-eclampsia parallels that of preterm birth, with placental dysfunction resulting from infection-mediated inflammation frequently playing a major role [4, 5].

Intrauterine infection accounts for 25–40% of preterm births with or without pre-eclampsia [5]. In fact, bacteria have been identified in the chorio-amnion of 80% of women who underwent cesarean section for preterm labor without a placental rupture, along with an associated inflammatory response in the amniotic fluid [6]. It is thought that the bacteria gain access to the amniotic fluid and chorion/placenta by ascending from the vagina, hematogenous dissemination from the placenta, accidental introduction during procedures, or retrograde spread from the fallopian tubes [6]. Indeed, bacterial vaginosis or imbalance in vaginal flora can result in a 1.5- to threefold greater risk for preterm birth [7, 8].

While local infection is the most proximate cause of inflammation, evidence from other diseases, e.g., rheumatoid arthritis and diabetes, implicates distant infection and systemic inflammation in the causal chain of events [9, 10]. Corroborating this, dysbiosis of the gut has been correlated with a heightened risk for adverse pregnancy outcomes [11, 12]. Indeed, maternal gut microbiomes associated with pre-term parturition demonstrate greater levels of oral bacteria than at-term controls [13].

On the other hand, the role of oral microbiota in the pathophysiology of pregnancy complications is not as well-defined. While a large body of evidence indicates that pre-term parturition and low birthweight are associated with maternal periodontal disease [14], a pathogen-rich subgingival microbiome [15], and oral bacteria in the placenta, the evidence remains equivocal. Additionally, a large-scale interventional study failed to demonstrate improvement in pregnancy outcomes following non-surgical periodontal therapy to reduce subgingival pathogen burden and inflammation [16]. However, these investigations employed cultivation-based approaches or molecular assays that targeted a small suite of species.

Sequence-based approaches, especially those with the capability to explicate the genome content and functionality of the microbiome, have revolutionized our knowledge of various human habitats by elucidating factors that facilitate the colonization and mobilization of these resident microbiota [17, 18]. Using these approaches, we are beginning to tease out the intricacies of the microbiome-gut-brain axis [19], the brain-endocrine-immune axis [20], and the gut-integument-microbiome axis [21]. These explorations are challenging traditional dogmas about the sterility of certain environments [22] and the circumscribed influence of local microbial communities [23].

Therefore, we aimed to combine whole-genome shotgun sequencing, real-time reverse transcriptase PCR, and source tracking with a case-control study design to (i) investigate whether the presence and levels of bacteria and bacterial genes in the placenta can discriminate between pregnancy outcomes, (ii) measure the impact of this genome content on the placental immuno-inflammatory response, (iii) use this system-scale profiling to derive salivary, subgingival, serum, and placental microbial signatures of pregnancy complications, and (iv) interrogate the subgingival and salivary microbiomes as potential sources of placental microbiota.

## Results

This analysis recruited 130 pregnant women with periodontal disease in the third trimester of pregnancy from over 9000 women who presented to the maternal and fetal medicine clinics. The groups were not significantly different in age (*p* = 0.1003, ANOVA), BMI (*p* = 0.1234, ANOVA), smoking status (*p* = 0.2212, chi-square), or clinical metrics of periodontal health (probing pocket depths and clinical attachment levels, *p* = 0.0750 and 0.2543 respectively, ANOVA). Both pre-term (PT) and pre-term complicated by pre-eclampsia (PTPE) groups delivered infants whose birth weight was significantly lower than the full-term (FT) group (*p* < 0.0001, ANOVA). Table 1 summarizes the clinical and demographic data.

**Table 1:**
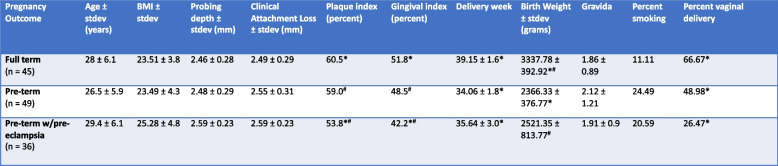
Summary of clinical and demographic data

Cells that share the same symbol are significantly different, *p* < 0.05. Plaque index shows the percentage of sites with plaque and gingival index shows the percentage of sites with bleeding on probing in each group calculated from the percentages in each woman

Quantitative PCR (q-PCR) revealed that placental and serum samples demonstrated significantly lower abundances of bacterial DNA when compared to subgingival and salivary samples (Table 2). A total of 1.47 billion total sequences derived from saliva, plaque, serum, and placenta samples were filtered down to 408 million bacterial sequences after removing human sequences. Serum samples had an average of 448,507 sequences (3,853 to 2,759,296), placental samples had an average of 285,334 (137,442 to 432,490), plaque samples had an average of 1,419,217 (569,856 to 3,487,658), and saliva samples had an average of 1,290,661 (283,084 to 5,188,072) after filtering out human reads.

**Table 2:** Mean DNA copy numbers in 2 µl for each sample type and group as estimated by qPCR

	Serum	Saliva	Subgingival plaque	Placenta	Placenta (PMA treated)
Pre-term (PT) (mean ± standard deviation))	39725 ± 9291^ab^	7885312 ± 120553	4999338 ± 65321	52825 ± 8812^abc^	23555 ± 4028^c^
Pre-term w/pre-eclampsia (PTPE) (mean ± standard deviation))	47671 ± 13092^ab^	7178412 ± 409932	4585285 ± 55382	43858 ± 7600^abc^	28703 ± 5192^c^
Full-term controls (FT) ((mean ± standard deviation))	37509 ± 14029	8072130 ± 187449	3712912 ± 20285	24993 ± 9492	13524 ± 3492

^a^Statistically different from saliva (*p* < 0.05, 2-sample *t*-test)

^b^Statistically different from subgingival plaque (*p* < 0.05, 2-sample *t*-test)

^c^Statistically different from full-term controls (*p* < 0.05, 2-sample *t*-test)

COGs that did not exceed a relative abundance of 0.01% were excluded from analysis as an unsupervised feature reduction technique, yielding 10007 functionally annotated genes (based on KEGG classification) were identified in the placenta, which contributed to 46 metabolic pathways. 9562 genes were identified in all three groups, while 445 were unique to one of the three groups. These sequences also represented 420 taxa across all samples (Supplemental Table S1).

### The placenta demonstrates a diverse microbial presence

We began our investigation by comparing placental samples to negative controls. In addition to strict sampling and preparation handling controls designed to eliminate environmental contamination (see “Methods” section), we prepared and sequenced “blank” samples containing only sequencing kit materials. Following these controls, we discovered that the placenta contains a suite of microbial genes that cannot be attributed to contamination from the environment, isolation kit, or sequencing (*p* = 0.014, PERMANOVA, Aitchison distance between kitome and placenta). The negative controls (“kitome”) contained 39 taxa, 34 which had three or fewer representative transcripts per sample (Supplemental Table S2). All these 39 taxa were identified in the placental samples and subtracted before further analysis.

In order to verify that the placental tissue contained whole microbial cells and not just microbial DNA, a portion of each placenta was treated with propidium mono-azide (PMA) and along with another un-treated portion, amplified for the 16S gene, and quantified (see “Methods” section). Both PMA-treated and untreated samples evidenced bacterial DNA, indicating that this community contained a mixture of bacterial cells and free nucleic acid material among other cellular constituents.

**Fig. 1:**
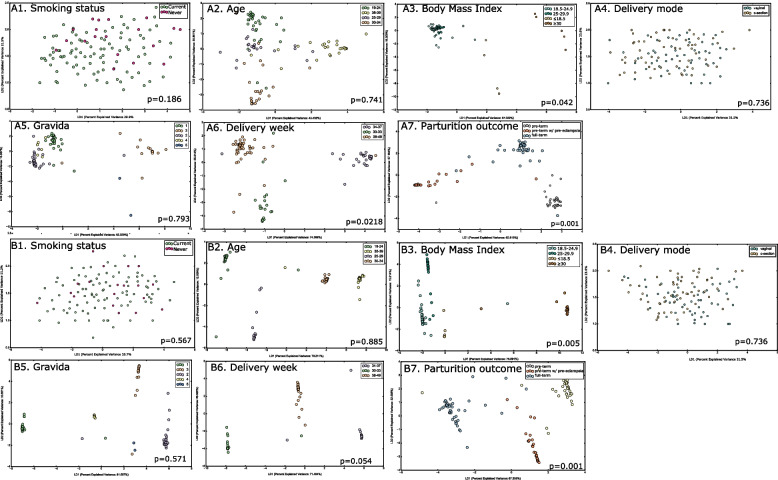
Discriminants of the placental microbial assemblages. Principle coordinate analysis (PCoA) of CLR-transformed taxa are shown in **A** and PCoA of CLR-transformed functional genes in **B**. In both panels, the data was mapped on smoking status (1), maternal age (2), body mass index (BMI) (3), delivery mode (4), number of pregnancies, i.e., gravida (5), delivery week (6) and parturition outcome (7). The significance of clustering was tested using PERMANOVA

We then investigated whether placental microbiota could be artifact induced by mode of delivery (Fig. 1A4, B4). Neither alpha (*p* = 0.655, Kruskal Wallis of Shannon diversity, Chao, ACE and Morisita Horn indices) nor beta diversity (*p* = 0.973, PERMANOVA and *p* = 0.282, PERMDISP, Aitchison distance of taxa and genes respectively) of the microbiomes differed between vaginal and caesarian modes of delivery. Comparison of the core microbial guild (species and genes in 80% of individuals in a group) identified 64 species and 67–80% of genes in common, while 12 species and 7% of functional genes could be attributed to vaginal delivery. *Prevotella *sp.* oral taxon 299*, *Leptotrichia *sp.* oral taxon 215, Neisseria elongata, Stomatobaculum longum, Eubacterium sulci,* and *Actinomyces *sp.* oral taxon 181* were identified only in vaginally delivered placentas while *Haemophilus haemolyticus, Leptotrichia wadei, Prevotella nigrescens, Gemella sanguinis, Fusobacterium *spp., and *Lautropia mirabilis* were unique to placentas delivered via c-section.

### Parturition complications and BMI are discriminants of bacterial assemblages in the placenta

To quantify bacterial presence in the placenta, we inducted identical volumes of placental tissue from all samples into the quantitative PCR as well as DNA processing and sequencing pipeline. On average, the DNA copy numbers as well as the number of sequences differed significantly between groups (*p* < 0.05, ANOVA, Table 2), with the highest number of sequences from placental samples in the PTPE group and the lowest from the FT group. To gain insights into the sources of variability in the placental microbiome, we integrated clinical and demographic metadata with Aitchison distance of the variance-stabilized abundances of genes and taxa. Class separation was quantified and visualized using linear discriminant analysis to investigate drivers of microbial presence in the placenta. BMI (*p* = 0.005, PERMANOVA) and pregnancy outcome (*p* = 0.001, PERMANOVA) emerged as significant discriminants of the placental metagenome, while maternal smoking status, maternal age, and gravida did not (Fig. 1A, B), corroborating previous evidence in the literature [24, 25].

Importantly, logistic regression revealed 32% higher odds of parturition complication (95% CI 2.8%, 81%) for every IQR increase in the Shannon diversity index after adjusting for maternal smoking status, maternal age, and gravida.

Comparing average within-group deviation from the group centroid in beta diversity (beta dispersion [26]) revealed that FT placentas exhibited the lowest dispersion, followed by PT. The microbiome of PTPE demonstrated the greatest within-group heterogeneity (*p* = 0.001, PERMDISP of Aitchison distances).

**Fig. 2:**
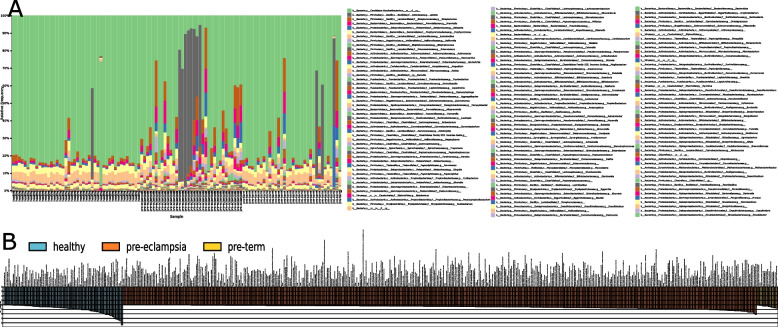
Differences in microbial community structure and function between full-term delivery, pre-term delivery, and pre-term delivery complicated by pre-eclampsia. Relative abundances of species-level OTUs are shown in each of the 130 women in **A**. **B** is a waterfall plot of the functional genes that were most likely to explain differences between classes using LefSe. Each bar represents the effect size (LDA) for a particular gene in a certain group. The length of the bar represents a log10 transformed LDA score. The data supporting this figure is available in Supplementary Table S1

Several recognized periodontal and endodontic pathogens were more abundant in PT and PTPE placental samples. For example, *Aggregatibacter actinomycetemcomitans* and *Enterococcus durans* were more prevalent in PT while *Granulicatella elegans*, *Tannerella forsythia*, *Treponema denticola*, *Prevotella intermedia*, and *Campylobacter gracilis* were more abundant in PTPE. Both sPLS-DA and LEfSe identified similar sets of species being discriminant of the respective sample groups, indicating that these differences were robust against analytical methods. The presence of *Bacillus subtilis* was indicative of full-term births, *Lactobacillis crispatus* was indicative of pre-term and pre-eclampsia, and *Corynebacterium matruchotti, Prevotella,* and *Tannerella* oral species were indicative of pre-eclampsia (Fig. 2A).

Bacterial genes encoding carbohydrate and protein metabolism, gram-positive cell wall components, and membrane transport were identified in all placental samples. However, genes encoding quorum sensing, necroptosis, ubiquitination, drug resistance, serine, proline biosynthesis, PTS transport system, peptidoglycan biosynthesis, and exosomes were enriched in both PT and PTPE groups (Fig. 2B).

A machine learning algorithm trained on the dataset was able to predict PTPE with 82% sensitivity and 87% specificity based on functional profiles and with 89% sensitivity and 88% specificity when using phylogenetic metrics. The classifier also predicted PT with 72% and 75% sensitivity and 81% and 69% specificity for functional and taxonomic profiles respectively.

### Specific microbial recognition pathways are associated with parturition complications

**Fig. 3:**
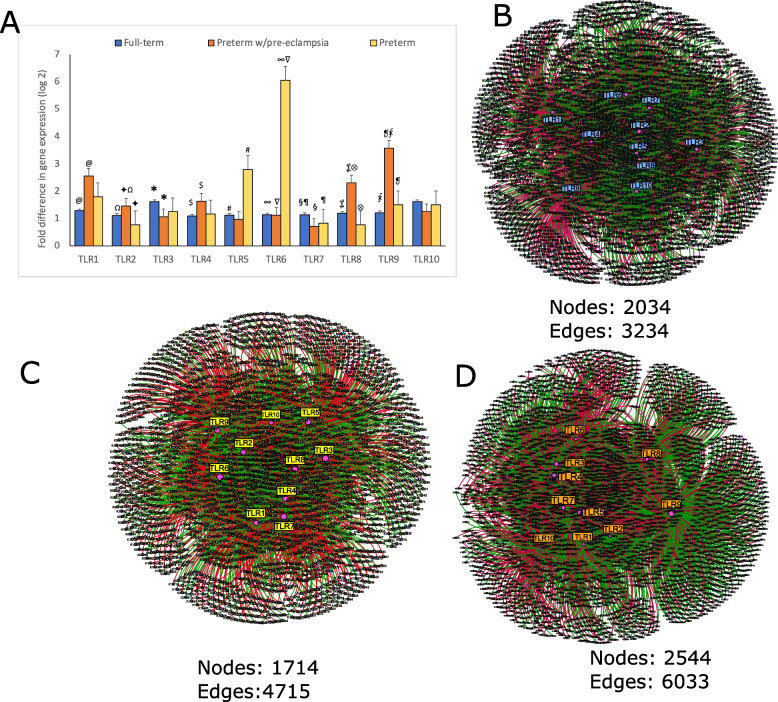
Bacterial signal recognition in the placenta. Levels of expression of 10 toll-like receptors (TLRs) in the placenta are shown in **A**. The *y*-axis represents log(2)-transformed concentrations. Bars with the same symbol are significantly different (*p* < 0.05, Dunn’s test). Co-occurrence networks between pattern recognition receptors and microbial genes in each group are shown in **B**–**D**. Full-term delivery is shown in **B**, pre-term delivery in **C,** and pre-term delivery complicated by pre-eclampsia in **D**. Each network graph contains nodes (circles) and edges (lines). Nodes represent TLRs and KEGG-annotated genes, and edges represent Spearman’s ρ. Edges are colored green for positive correlation and red for negative correlation. Only significant correlations (*p* < 0.05, *t*-test) with a coefficient of at least 0.80 are shown

Quantitative reverse-transcriptase PCR revealed significant over-expression of TLR5 and TLR6 in the PT group when compared to the FT group. Moreover, TLR1, TLR2, TLR4, TLR8, and TLR9 were significantly upregulated in the PTPE group when compared to the FT group, while TLR5, 6, and 9 were overexpressed in the PT group in comparison to the FT group. TLR7 was significantly downregulated in both PT and PTPE groups when compared to FT (Fig. 3A). These patterns of TLR expression point to the recognition of signals from microbial nucleic acids in PTPE and a preponderant response to pyogenic bacteria in the PT group.

We then used graph theory to verify that the TLR response was indeed attributable to bacterial triggers. TLRs were highly networked with bacterial genes, with the PTPE group demonstrating the greatest co-occurrence (6035 edges and 4017 nodes), the highest betweenness centrality and modularity followed by the PT and FT groups (Fig. 3B–D). In the PTPE group, genes encoding gram-positive cell wall components and gram-negative membrane-associated proteins, membrane transporters, peptidoglycan biosynthesis, and enzymes within the protein and amino acid metabolism pathway were network anchors, while membrane transporters, peptidoglycan biosynthesis, and iron metabolism anchored the PT network.

Together, the high receptor signals and dense metagenome-TLR network topology point to bacterial nucleic acids as well as cellular components of gram-negative, gram-positive, and pyogenic bacteria as inflammatory triggers in placental tissue.

### Placental microbiota has a systemic origin

We used a Bayesian analysis method (SourceTracker) to investigate the extent to which the placental microbiota could be traced to the serum versus other sources. One hundred percent of the placental samples contained microbiota that could be traced to the serum.

Even more importantly, in the FT group, 82% of the placental microbial community could be traced to the serum, while 73% and 81% of the community in the PT and PTPE groups demonstrated serum origins. We repeated the analysis using the core placental species (species present in ≥ 80% of individuals in each group) and arrived at the same result, suggesting that placental microbiota have a systemic/serum origin and do not merely represent transient bacterial cells or nucleic acid material.

### Serum analysis provides evidence of hematogenous spread from the oral cavity

We then used the same analysis to track serum microbiota to oral sources. We discovered that, indeed, oral bacteria translocated into the systemic circulation in all subjects, and that > 70% of serum microbiota were sourced from the salivary microbiome. Subgingival plaque contributed to 1.4% of serum bacteria in FT, 12% in PT, and 6.8% in PTPE groups (*p* = 0.001, ANOVA). Approximately 15% of the serum microbiota was attributable to a non-oral source in FT and PT groups but only 3% in PTPE (*p* = 0.007, ANOVA).

The most frequently identified oral taxa in serum samples included *Streptococcus *spp., *Corynebacterium matruchotii*, *Fusobacterium nucleatum*, and *Granulicatella elegans*. Together, these contributed to 62% of the bacterial abundance in serum, however, their abundances differed significantly between groups, with PTPE demonstrating significantly higher levels of *F.nucleatum* and *C. matruchotii* when compared to FT, while *F.nucleatum* and *G.elegans* were more abundant in the serum of PT group when compared to FT. Moreover, *Atopobium minutum,* a *Haemophilus* phage, and *Lactobacillus* phage were uniquely identified in the FT serum, while *Lactobacillus coleohominis* and *Aggregatibacter* phage were unique to PTPE and *Eubacterium limosum* and *Haemophilus aegyptius* were identified only in the PT serum.

**Fig. 4:**
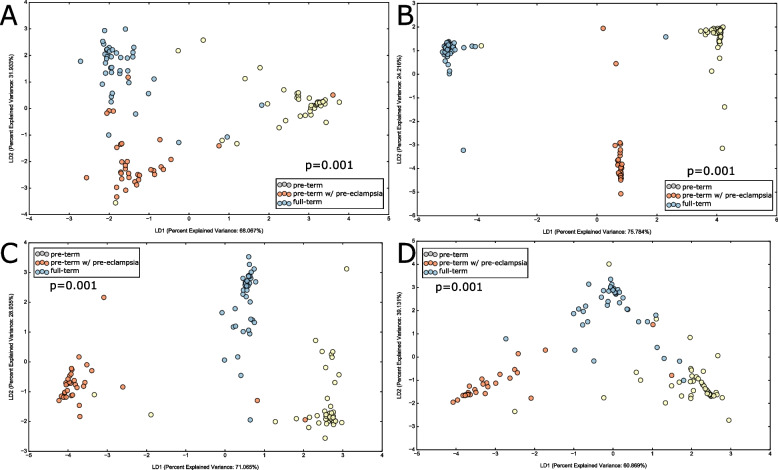
Differences in salivary and subgingival microbial community structure and function between full-term delivery, pre-term delivery, and pre-term delivery complicated by pre-eclampsia. Linear discriminant analysis (LDA) of CLR-transformed salivary functional genes is shown in **A**, subgingival genes in **B**, salivary microbial taxa in **C,** and subgingival microbial taxa in **D**. The microbial profiles of subjects clustered by delivery type creating three statistically significant clusters (*p* = 0.001, MANOVA/Wilks)

**Fig. 5:**
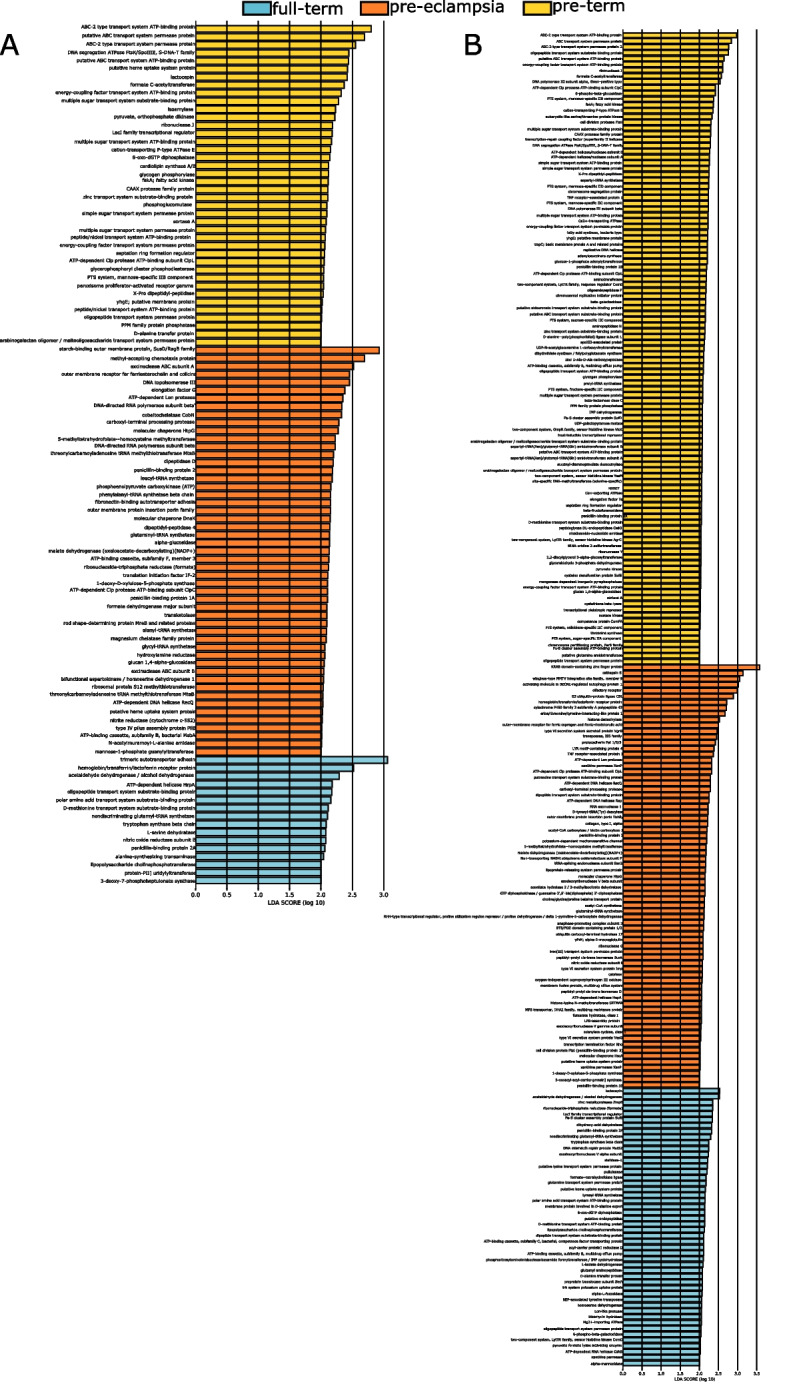
Waterfall plot of subgingival (**A**) and salivary (**B**) functional genes that were most likely to explain differences between classes using LefSe. Each bar represents the effect size (LDA) for a particular gene in a certain group. The length of the bar represents a log10 transformed LDA score

We then investigated if these differences in serum were reflected in salivary and subgingival microbiomes using linear discriminant analysis (LDA) to examine group separation. Significant clustering of the salivary and subgingival microbiomes by parturition complication was evident (*p* = 0.001, PERMANOVA, Aitchison distance, (Fig. 4)), with higher abundances of *Prevotella *spp., and* Fusobacteria *spp. in the PTPE saliva, and *Streptococcus *spp. and *Actinomyces spp.* in the PT saliva when compared to the other two groups. Similarly, *Provetella *spp.,* Treponema *spp., and *Porphyromonas *spp. were over-represented in the subgingival microbiome of the PTPE group when compared to FT and PT. In both saliva and plaque, genes encoding iron transport, exosomes, adhesion, quorum sensing, lipopolysaccharide biosynthesis biofilm formation, chaperones and ubiquitin, and steroid degradation were elevated in the PTPE group when compared to FT, while COGs corresponding to exosome, lysosome, quorum sensing, adhesion, membrane trafficking, chaperones and ubiquitin and biofilm formation were enriched in the PT group over the FT group (Fig. 5).

## Discussion

Our knowledge of the placental environment is continually evolving; beginning with the “sterile womb hypothesis” [27], to the identification of a low biomass yet distinct “microbiome” [25, 28, 29] that resembled the oral microbiome, to studies which posit that the “placental microbiome” is a contaminant from laboratory reagents or an artifact created by forces of labor and delivery [30]. When we combined whole genome shotgun sequencing of a carefully curated set of placental, serum, salivary, and subgingival plaque samples with quantitative PCR of pattern recognition receptor signals and graph theoretic based on social media networking algorithms, we discovered not only a microbial presence in the placental tissue that could be traced to the oral cavity via the systemic circulation but also a florid upregulation of receptors that recognize these microbial motifs.

Recognizing that bacterial presence in the placenta does not denote colonization [31] and that a cross-sectional investigation is not designed to distinguish between transient members (allochthonous constituents) or stable colonizers (autochthonous community) [17, 32], we used multiple complementary strategies and stringent controls to interrogate the placental microbiome. We (a) sampled only those women who underwent scheduled c-section prior to the onset of labor, and excluded unscheduled c-sections (starting as vaginal delivery but ending up with c-section) to control for labor-induced placental blood flow as an explanation of bacterial presence in the placenta [30, 33], (b) minimized contamination during sample collection, processing, and DNA isolation, (c) examined the core metagenome to minimize the effect of allochthonous species and genes on the analysis (borrowing from the strategy initially used by the Human Microbiome Project), (d) investigated mode of delivery as a potential source of contamination, and (e) controlled for PCR and sequencing artifacts by using positive and negative controls.

Another concern arising from DNA-based approaches is the inability to differentiate between intact bacterial cells versus free-floating nucleic acid (relic DNA) [18]. We used a two-pronged approach to navigate this question. As a first step, we tested the hypothesis that bacterial presence in the placenta was due to cellular debris by treating the samples with PMA to remove relic DNA. Both PMA-treated and untreated samples produced strong bacterial signals following DNA amplification, indicating that the placental metagenome was derived from a mixture of intact cells and bacterial nucleic acid. As a second step, we used network analysis to investigate TLR-microbial interactions. Bacterial DNA is sensed by a subset of TLRs that reside in endosomal compartments (intracellular TLR), specifically, TLR3 (dsRNA), TLRs 7 and 8 (ssRNA), and TLR 9 (hypomethylated cytosine-phosphate-guanine (CpG) DNA) [34, 35]. On the other hand, TLRs 1, 2, 4–6, and 10 localize to the cell surface and recognize pathogen-associated microbial patterns (PAMPs) such as lipids, lipoproteins, flagellin, peptidoglycan, lipoarabinomannan etc. [36]. The amplitude of signals we recorded from all 10 TLRs strongly bolsters the evidence that a combination of bacterial cells as well as bacterial genetic material is associated with pregnancy complications.

The most striking finding was that the placenta is not free of microbial presence even in states of health, corroborating previous studies using cultivation-independent approaches [25, 37]. However, in the present investigation, not only were significantly more bacterial sequences identified in the placenta of PTPE and PE groups than FT, but specific microbial assemblages were also associated with each pregnancy outcome. Furthermore, different combinations of pattern recognition receptors were upregulated in each group, pointing to different types of bacterial triggers. Corroborating this, significantly higher numbers, and different permutations, of TLR-microbial gene interactions were evident in the PTPE and PT groups in comparison to the FT group. Together, the data point to a state of homeostasis between a low biomass microbial community and the immuno-inflammatory machinery in the healthy placenta. The data also indicate that this immuno-tolerant system can be overwhelmed by higher bacterial loads or pro-inflammatory bacterial triggers or both. In addition, the evidence points to the fact that this disruption is associated with adverse pregnancy outcomes. In this context, we must make a reference to our finding that *B. subtitis* was identified as a discriminator of full-term births. While it is possible that this is an artifact of the database, evidence is emerging from recent studies that this organism is a normal commensal of the human gastrointestinal tract [38]. Moreover, prebiotic supplementation with this organism in late gestation has been shown to exert beneficial reproductive outcomes in animals [39, 40]. While we do not have data about prebiotic usage in our subjects, these lines of evidence might suggest that *B. subitis* is not necessarily an artifact, but that probiotics for human reproductive health are a promising line of investigation.

We also find evidence of a strong pro-inflammatory pathology underlying pre-term delivery, either with or without pre-eclampsia. Immune tolerance towards paternal and fetal proteins that leak into the maternal circulation is critical to successful gestation [41]. It is now recognized that inflammation revokes this immune privilege, which creates a downstream cascade of events resulting in pregnancy complications [42]. Of particular interest to us is TLR9, which demonstrated ~ 100-fold higher gene expression in PTPE and ~ eightfold higher expression in the PT group in comparison to the FT group. TLR9 has evolved to sense CpG-rich hypomethylated DNA, which is largely found in bacterial, mitochondrial, and fetal DNA [43]. Emergent evidence identifies TLR9 as a key player in blood pressure regulation [44] and TLR9-mediated inflammation is implicated in the causal chain of pre-eclampsia [45]. In support of this, a concomitant downregulation of TLR7 (capable of downregulating expression of TLR9 [46]) was observed in both the PT and PTPE groups. Both TLR7 and 9 are responsible for recognizing and responding to microbial nucleic acid in the endosome, however, TLR7 plays an additional role in inhibiting CpG-ODN induced IFNα production from plasmacytoid dendritic cells and B cells following TLR7/TLR9 co-stimulation [47]. This inverse link between the two receptors, along with the remarkably amplified signal, suggests an important role for TLR9 in driving pregnancy outcomes, warranting further investigation.

While local inflammation could be the underlying cause of this hyperinflammatory signature, two key findings argue against it. The first is that all subjects were negative for bacterial vaginosis or other local infections. The second is that 70–82% of the placental microbiota in each woman was a complete subset of her serum bacteria. Bacterial translocation to the serum is well established, as is the link between serum bacterial load and hypertension [48]. Plasma LPS levels are a known predictor of future sustained hypertension, which cannot be explained by other cardiometabolic risk factors [49, 50]. In support of this argument, we observed the highest serum bacterial load as well as the largest numbers of serum-derived bacteria and bacterial genes in the placentae of the PTPE group, which consists of hypertensive women. It is possible that either specific species in the serum or high abundances of circulating bacteria predispose these women to hypertension, and during pregnancy, the altered vascular dynamics create a scenario favorable to seeding the placenta with these organisms. Alternatively, the pathological hemodynamics created by hypertension could be the underlying factor in increased systemic bacteremia as well as the homing of these organisms to the placenta. It is beyond the scope of this study to investigate this hypothesis, however, exploring the inter-relationship between hypertension and bacterial dissemination into circulation will be important to explicate the link between bacteremia and several non-infectious systemic diseases.

Our data also suggest that the oral microbiome is a predominant source of bacteria that translocate via the serum to the placenta, corroborating multiple human and animal studies [25, 51, 52]. The systemic circulation is seeded by oral bacteria during daily activities such as chewing, brushing, and flossing [53, 54], therefore it is not surprising to find oral bacteria in highly vascular niches. Pregnancy induces changes in the oral mucosal barrier and vasculature [55], further facilitating the egress of bacteria from this ecosystem into the circulation. We have previously demonstrated that the pregnancy-associated oral microbiome is enriched for species belonging to the genera *Pseudomonas, Acidovorax, Enterobacter, Enterococcus, Diaphorobacterium,* and *Methylobacterium *[56]. Corroborating and expanding on previous evidence, we now find evidence to suggest that specific salivary and subgingival microbial profiles are associated with dissemination to the serum. Of note are genes encoding biofilm formation, chaperones and ubiquitin, iron transport, and adhesion. It is important to note that all of our subjects demonstrated gingivitis or early periodontitis, suggesting that integrating oral healthcare into pre- and ante-natal care is critical.

In summary, within the limits of a cross-sectional analysis, we find evidence to support the theory that infection-mediated inflammation underlies the etiology of pregnancy complications. While oral bacteria translocate to the placenta during all pregnancies, they exert a powerful, detrimental effect on the placental system in certain subjects, altering the immunotolerance of the mother, and triggering a signaling pathway capable of inducing placental vascular pathology. Two possible mechanisms appear to underlie this phenomenon: systemic hypertension and oral microbial signatures rich in genes encoding iron transport, adhesion, biofilm formation, and lipopolysaccharide. To the best of our knowledge, this investigation provides the first evidence that implicates TLR recognition of oral microbial signatures in obstetric syndromes and begins to explore potential mechanisms that underlie this pathology.

## Methods

### Study approval

Approval for this study was obtained from the Office of Responsible Research Practices at Health Science University Tepecik Education and Research Hospital (IRB protocol number 40465587-28) and the study was conducted between January and December 2018 in accordance with approved guidelines. Written informed consent was received from all subjects prior to participation.

### Subject selection and recruitment

One hundred thirty women in their third trimester of pregnancy who fulfilled the following inclusion and exclusion criteria were recruited following written informed consent, and comprehensive clinical periodontal examination. The inclusion criteria were age between 18–34 years, clinical periodontal diagnosis of gingivitis or Stage 1 periodontitis (defined by probing depths ≤ 5 mm, bleeding index (BOP) > 30% and clinical attachment loss < 2 mm), systemic health (defined as ASA (American Society of Anesthesiologists) I or II) and pregnancy with a single fetus. Plaque index and gingival bleeding index were evaluated dichotomously as present or absent and percentages of sites with plaque and bleeding were calculated for each woman, and then mean percentages were calculated for each group. Exclusion criteria included body mass index (BMI) of ≥ 30, history of pre-eclampsia in previous pregnancy, history of cervical cerclage and amniocentesis in current pregnancy, oligohydramnios, polyhydramnios, stillbirth history, fetus complicated with chromosomal abnormalities, HIV or vaginal infection, preterm premature rupture of membranes (PPROM), malignancy, GDM, any acute or chronic infectious or inflammatory disease, sepsis, hemoglobulin ≤ 10, thrombocytopenia (platelet count < 50 × 10^9^/L), thrombocytosis (platelet count > 50 × 10^11^/L), uterus abnormalities, hypertension, coagulation disorders, deep vein thrombosis or use drugs that affect platelet function such as acetylsalicylic acid. Patients with significant obstetric or medical complications, unreliable maternal and fetal status information, clinical chorioamnionitis, placental abruption, significant antepartum hemorrhage, and intrauterine growth restriction (10% of estimated standard birth weight below for gestational age), controlled or uncontrolled diabetes, those using immunosuppressant medications, bisphosphonates, or steroids, antibiotic therapy or oral prophylactic procedures within the preceding 3 months, and fewer than 20 teeth in the dentition were also excluded. Sample size was estimated based on the probability of least an 80% chance of detecting clades of bacterial genes that differed in abundance by more than 2% [57]. Forty-five women delivered full-term (FT), 49 women without pre-eclampsia delivered pre-term (PT), and 36 women who delivered preterm also had pre-eclampsia (PTPE).

### Sample collection

Saliva, subgingival plaque, serum, and placental samples were collected at the time of delivery. Subgingival plaque samples were collected and pooled from 15 sites on 6 maxillary and mandibular anterior teeth using sterile endodontic paper-points (Caulk-Dentsply, Milford, DE, USA). Prior to subgingival plaque sampling, supragingival plaque was removed gently using sterile Gracey curettes while paying attention not to induce gingival bleeding. Saliva samples were collected by having all the participants expectorate into collection tubes for 3 min. Serum was collected by venipuncture of the right ante-cubital fossa. Placental dissection was done immediately after delivery. The covering decidua basalis on the maternal side of the placenta was removed, and 3 × 3 cm of placental tissue pieces were taken from different sites along the placenta and placed in sterile calcium/magnesium-free phosphate-buffered saline (PBS) (Gibco, UK). Care was taken to avoid contamination with the chorioamnion. The tissue was repeatedly washed with PBS to remove contaminated blood, and then a 1-mm^3^ sample was minced from each placenta and snap-frozen in separate tubes. Care was taken not to form any blood clots or fibrous tissue. The placenta and plaque samples were stored at − 20 °C until sample processing, and the serum and saliva samples were divided into 1 ml aliquots and freeze-dried in a vacuum freezer before storage at − 20 °C.

### DNA isolation, biomass estimation, and metagenomic sequencing

DNA was isolated using the MagMAX Total Nucleic Acid Isolation Kit (Applied Biosystems) as previously described [58]. Briefly, the subgingival plaque samples were prepared by adding 100 μL phosphate-buffered saline (PBS) to the paperpoints and vortexing for 30 min at room temperature, incubating at 43 ºC hot water bath for 10 min, then incubating at 4 ºC overnight. The placental samples were prepared by adding 100 μL of PBS to 1 g of sample and vortexing for 30 min at room temperature, incubating at 43 ºC hot water bath for 10 min, then incubating at 4 ºC overnight. The serum and saliva samples were prepared by adding 100 μL of PBS to the lyophilized sample, vortexing for 30 min, and incubating overnight at 4 ºC. One hundred microliters of solution for each of the samples was then used for DNA isolation following the manufacturer’s protocol. The samples were isolated in a fume hood that had been cleaned with 70% ethanol and sterilized by UV light for 30 min before isolation. Libraries were generated using the NEBNext® UltraTM II FS DNA Library Preparation Kit for Illumina (New England Biolabs, Inc.). 100 ng of the sample was used as input. DNA was fragmented to 100–250 bp. Adapters were then ligated to the fragments. Twelve unique adapters were used so that 12 samples could be pooled together during sequencing. Size selection was not performed, however, the adapter-ligated samples were cleaned before proceeding with PCR enrichment. Six cycles of PCR were completed before cleaning and pooling the samples. Pooled libraries were sequenced on the Illumina HiSeq4000 platform using 150 bp paired-end chemistry.

Two microliters of DNA were used for quantitative real-time PCR. The samples were analyzed for total bacteria using a previously described methodology [59]. Briefly, 400 pg of DNA was used as a template in a 25-μl reaction containing 0.5 U of Platinum *Taq* polymerase, buffer containing 50 mM KCl, 10 mM Tris–HCl (pH 8.8) 5 mM MgCl_2_, a 0.2-mM concentration of each deoxynucleoside triphosphate, 10 ng of yeast tRNA, 0.8 μM concentrations of forward and reverse primers CCTACGGGDGGCWGCA and GGACTACHVGGGTMTCTAATC, 100 nM probe (6FAM-CAGCAGCCGCGCGGTA), and 60 nM Rox reference dye. Amplification and detection were carried out in a 384-well format on a QuantStudio 12 K Flex instrument (Applied BioSystems). Bacterial DNA concentrations were computed by comparison to standard curves generated from known amounts of 4 bacterial species.

### Quality control

DNA isolation kits were newly opened immediately prior to sample prep and used exclusively for the present investigation. For each batch of sample preparation and DNA isolation, a negative control was processed alongside the samples by carrying out all steps in the DNA isolation protocol with the exception that sterile paper points were used instead of the paper points containing samples. All samples were sequenced in two runs, and samples were randomly assigned to each run to minimize batch effects. Both positive (defined culture) and negative (no template) controls were used. Replicate sequencing was carried out for two samples in each batch, and the replicates showed good reliability across the 5 batches, with a coefficient of variability ranging from 0.26 to 1.3% for alpha diversity of taxonomy, and 3.4 to 6.3% for predominant functions (carbohydrate metabolism, respiration, and virulence, disease, and defense).

To investigate if the placenta contained intact bacteria or nucleic acid material, a portion of the placental tissue was treated with propidium mono-azide (PMA) [60], and DNA isolation was carried out using the same protocol described above. Both PMA-treated and untreated samples were amplified using 40 cycles of PCR with universal primers targeted to the 16S rRNA gene (*805R (5′-GAC TAC HVG GGT ATC TAA TCC-3′) and 341F (5′-CCT ACG GGN GGC WGC AG-3)*. The PCR was replicated, and amplicons were visualized using gel electrophoresis with ethidium bromide staining. Additionally, both PMA and non-PMA-treated samples were subject to quantitative PCR as described above.

### Metagenomic sequence analysis

Sequences were quality filtered, and screened for human DNA using Sickle and the short-read alignment tool, Bowtie [61]. The phylogenetic profiles were assigned using Kraken [62] trained on the Human Oral Microbiome Database (HOMD) [63]. Prodigal was used for coding sequence (CDS) prediction, and genes were aligned against the NCBI nonredundant database of proteins using DIAMOND [64]. The biological pathways and protein functional categories were determined by assignment to the Kyoto Encyclopedia of Genes and Genomes orthology (KEGG) [65], and the Virulence Factor Database (VFDB) [66] using MEGAN [67].

### Quantification of placental toll-like receptor (TLR) gene expression

Total RNA was harvested from 2 gms of placental samples and reverse transcriptase quantitative PCR (qPCR) was used to measure gene expression. cDNA was generated from 500 ng of RNA using the SuperScript™ VILO™ Master Mix (ThermoFisher Scientific (Waltham, MA, USA)) according to the manufacturer’s protocol. Briefly, a reaction mixture containing 4 μL of 5X SuperScript Enzyme Mix, 500 ng of total RNA, and nuclease-free water was incubated using the following cycling parameters: 25 ˚C for 10 min, 42 ˚C for 60 min and 85 ˚C for 5 min. The cDNA mixture was then diluted 1:10 and qPCR was performed using the following conditions: 95 ˚C for 20 s, 95 ˚C for 1 s, and 60˚ for 20 s for 40 cycles. Ct values were generated for 10 TLRs and GAPDH (housekeeping gene). No-template controls were included in all reactions. Primers and probes for all target genes were purchased from ThermoFisher Scientific.

### Statistical analysis

Principal Coordinate Analysis (PCoA) was used for dimensionality reduction and visualization of microbial data. The significance of group-wise clustering in the Principal Coordinate Analysis (PCoA) was interrogated with a permutational multivariate analysis of variance (PERMANOVA) using the R package vegan [68]. Sparse partial least squares discriminant analysis (sPLS-DA) using the mixOmics R package [69] and linear discriminant analysis effect size (LEfSe [70]) were used to determine the drivers of the differences. LefSe uses a non-parametric factorial Kruskal–Wallis sum-rank test to identify significantly differential abundances between groups, the unpaired Wilcoxon rank-sum test to estimate biological, and LEfSe uses LDA to estimate the effect size of each differentially abundant gene/taxa. PhyloToAST [71] and QIIME2 [72] were used for data visualization and statistical analyses.

Bayesian analysis (SourceTracker [73]) was used to trace the sources of the serum and placental microbiomes. Datasets were filtered to remove genes/taxa that were not present in at least 1% of samples. Default parameters (rarefaction depth 1000, burn-in 100, restart 10, alpha (0.001), and beta (0.01) dirichlet hyperparameter) were used for analysis.

The ability of genes and species to discriminate between groups was examined using a machine-learning algorithm (RandomForest package in R). Two-thirds of the dataset was used to train the classifier, which was tested on the remaining data (1000 trees/tenfold cross-validation). For all iterations of the test a ‘confusion table’ was created for each of the exposures based on the number of correctly classified and misclassified samples, and this data was used to compute sensitivity and specificity. The robustness of the classifier was evaluated using ROC curves (ROCR package in R).

Co-occurrence networks were created between abundances of functionally annotated bacterial genes and TLR transcripts for each group. To decrease the occurrence of spurious associations due to rare taxa, co-occurrence networks were computed only on the core taxa [74]. JMP (SAS Institute Inc., Cary, NC, USA) was used to calculate pairwise correlations; significant co-occurrences (defined as Spearman’s rho > 0.75 and *p* < 0.05 (*t* test of rho)) were imported into Networkx [75] to create the graph structures, and Gephi[76] to visualize and label the graphs. Betweenness centrality was calculated using Python package ‘Networkx’. Robustness of clustering was examined using an algorithm incorporating betweenness centrality, differential abundances, and frequency of occurrence in a group as described before [56].

Differences in the expression of TLR genes were tested using ANOVA with correction for multiple comparisons (Tukey HSD). Normalized gene expression data was inducted into the analysis.

Cells that share the same symbol are significantly different, *p* < 0.05. Plaque index shows the percentage of sites with plaque and gingival index shows the percentage of sites with bleeding on probing in each group calculated from the percentages in each woman.

### Supplementary Information

**Additional file 1: Supplemental Table S1.** Data supporting Figure 2. Relative abundances of genes and taxa in each group.

**Additional file 2: Supplemental Table S2.** Genes and taxa identified in the negative controls.

## Data Availability

Sequence data has been uploaded to the Sequence Read Archives (SRA) of the National Center for Biotechnology Information (NCBI) database with the following identifier: PRJNA934596.
